# Fluctuating paraplegia secondary to spinal epidural lipomatosis: a case report

**DOI:** 10.3389/fsurg.2025.1533158

**Published:** 2025-04-11

**Authors:** Malak El Ayssami, Carmen Adem, Christian Attieh, Mounir Khoury

**Affiliations:** ^1^ Department of Neurology, Saint Georges Hospital, Balamand University, Beirut, Lebanon; ^2^ Department of Radiology, Saint Georges Hospital, Balamand University, Beirut, Lebanon; ^3^ Institut de la Colonne Vertébrale et des Neuro Sciences (ICVNS), Paris, France

**Keywords:** lipomatosis, myelopathy, decompression, epidural - thoracic, obese

## Abstract

Spinal epidural lipomatosis (SEL) is a rare condition characterized by excessive growth of adipose tissue in the epidural space, leading to spinal cord or nerve root compression. We present a case of a 37-year-old obese male who developed fluctuating paraplegia secondary to SEL. The patient initially presented with ascending weakness, which rapidly progressed to paraplegia, accompanied by sensory deficits and urinary incontinence. MRI revealed SEL with associated spinal cord compression. Although initially planned for decompressive surgery, the patient showed partial recovery, delaying the procedure. After laminectomy and excision of the lipomatosis, the patient demonstrated significant motor improvement, although mild sensory deficits persisted. Histopathological analysis confirmed the presence of adipocytes. This case highlights the importance of recognizing SEL, especially in patients with fluctuating neurological symptoms. Treatment strategies include weight management and surgical decompression, with a favorable prognosis for those undergoing timely intervention. Early diagnosis through MRI and appropriate management is key to improving outcomes.

## Introduction

Spinal epidural lipomatosis (SEL) is a rare but well-recognized entity. It is defined as an abnormal excessive growth of the normally present adipose tissue in the epidural space causing compression and dysfunction of the underlying nervous system. It is typically diagnosed using magnetic resonance imaging, which in severe cases reveals the Y sign of the dural sac (1). In 1975, Lee et al. described the first symptomatic epidural lipomatosis in a patient post-renal transplantation (2). In the subsequent cases reported, exogenous steroids use had the highest associative factor with the diagnosis of SEL, while obesity came second (3). Here we report a case of SEL uncommon because of the fluctuations of its clinical features, in a 37-year-old male obese patient who was successfully treated with decompressive surgery.

## Case presentation

A 37-year-old male patient presented with a one-month history of ascending weakness in his right lower limb, accompanied by several years of pressure sensation in the rectum. This weakness progressed rapidly within three days to paraplegia. Additionally, the patient experienced two episodes of urinary incontinence during the same week as his presentation.

Neurological examination revealed normal gross and pinprick sensation in bilateral upper extremities, with 5/5 motricity, but marked decrease in pinprick sensation and point discrimination starting from the level of the Xiphoid and downwards. The examination also indicated absence of abdominal reflex and cremasteric reflex, with brisk knee jerk reflex and Achilles reflex, and clonus in bilateral lower limbs. There was 1/5 motricity in bilateral proximal and distal muscles of the lower limbs. Proprioception was absent bilaterally in the lower limbs, along with dorsiflexion of the toes bilaterally. The patient had a BMI of 32 kg/m^2^ (Weight: 108 kg; Height: 183 cm) at the time.

**Figure 1:**
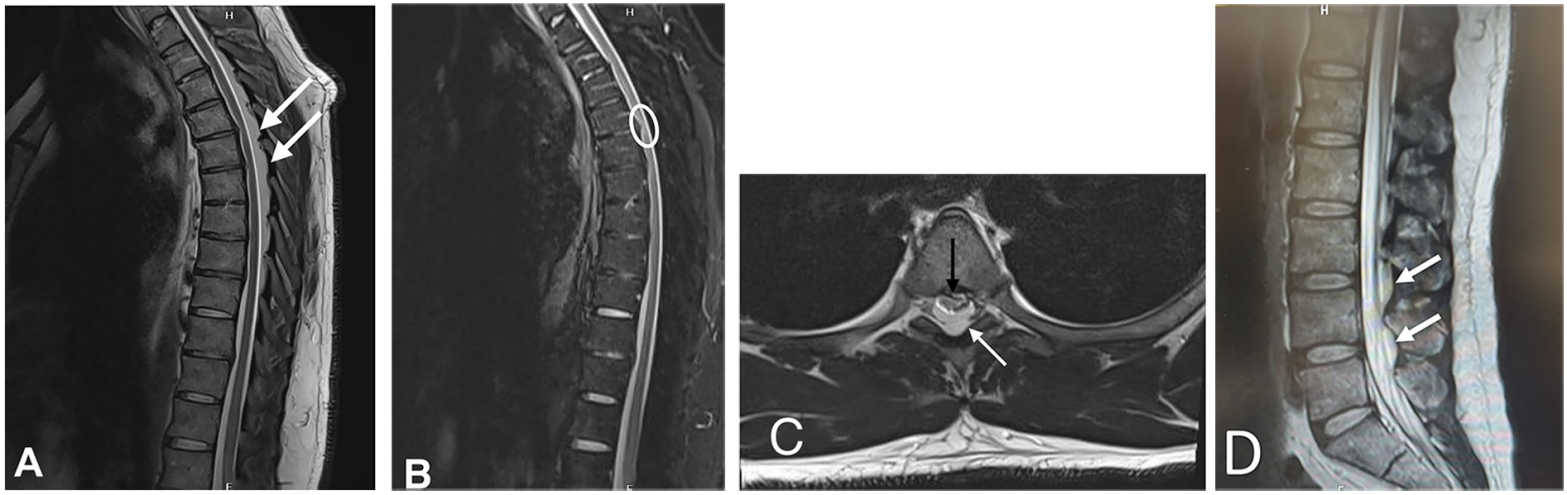
Thoracic spinal magnetic resonance Imaging. T2 sagittal sequence shows significant stenosis of the spinal cord by epidural adipose tissue (arrow, **A**), confirmed to be adipose tissue based on signal dropout on T2 sequence with fat suppression **(B)** with hyperintensities seen in the left aspect of the spinal cord at T4/5 and T5/6; the epidural adipose tissue again noted in the axial aspect T2 sequence (White arrow, **C**) with marked spinal canal stenosis (Black arrow, **C**) **(D)** Sagittal sequence showing a non-compressing lumbar epidural lipomatosis (White arrows).

Spine MRI with gadolinium (Figure 1), performed upon his arrival at the ER, revealed spinal epidural lipomatosis resulting in compression of the thoracic spinal cord. Additionally, there were a few tiny T2 STIR signal intensity areas in the left aspect of the spinal cord at T4/5 and T5/6, indicative of myelopathy at this level. Mild spinal canal narrowing was observed due to a few posterior disc protrusions at T4/5 and T7/8 (Figure 1B) The spine MRI also showed adipose tissue deposition in the lumbosacral area without compressing the nerve roots (Figure 1D).

Brain MRI showed no intracranial abnormalities and laboratory tests were also within normal limits.

Due to the severity of his neurological symptoms, the patient was scheduled for a laminectomy on the same day of admission. However, later that night, the patient experienced partial recovery with improved motricity to 3/5 bilaterally in his lower limbs. Patient was also able to ambulate with assistance, albeit with an ataxic gait. The remainder of the physical examination remained unchanged. Thus, excision of the epidural lipomatosis was postponed and performed on the following day.

Post-operatively, the patient showed remarkable recovery in motor power in his bilateral lower limbs, although his gait remained mildly ataxic with persistent sensory deficits from his initial presentation. He received pulse steroid therapy with 1 g Solumedrol IV over 5 days, followed by a Prednisone taper from 60 mg for 10 days upon his discharge. Histopathology showed adipocytes.

Three months after the surgery, the patient reported significant improvement in his condition. He described a smooth recovery process and mentioned that he had nearly returned to his normal daily activities. He can walk without assistance, exhibiting a mild ataxic gait that is not debilitating, and has no bladder or bowel dysfunction. The patient expressed gratitude for the positive changes in his quality of life and the progress he had made since the surgery.

## Discussion

Spinal epidural lipomatosis (SEL) is characterized by the abnormal accumulation of normal adipose tissue within the spinal canal, leading to compression of the spinal cord or nerve roots. SEL can be classified into two forms: the idiopathic (primary) form, typically associated with obesity, and the secondary form, which is linked to conditions such as endocrinopathies or steroid use (3). Recent cases of SEL have predominantly been associated with obesity, followed by prolonged use of exogenous steroids. Additionally, a number of non-steroid-related cases have been reported, including those connected to conditions such as Cushing's disease/syndrome, hypothyroidism, and pituitary prolactinoma. Some idiopathic cases have also been documented. Recent studies suggest that SEL may be considered a manifestation of metabolic syndrome, often seen in conjunction with increased BMI, abdominal circumference, and visceral and liver fat deposits (4).

Patients with SEL present with a wide range of symptoms, from being entirely asymptomatic to exhibiting muscle weakness, sensory loss, abnormal reflexes, or bowel/bladder dysfunction (5, 6). Some cases have shown unusual fluctuations in symptoms, which have been documented in only a few instances (7). While the condition generally has a gradual onset with slow progression of neurological features over months, the rapid onset seen in our case is atypical (8). The progression of myelopathy is thought to result from both direct mechanical compression of neural structures and indirect vascular dysfunction, such as compression of epidural blood vessels, leading to venous engorgement (7).

MRI remains the gold standard for diagnosing and grading SEL. The thoracic and lumbar regions are most commonly affected (1). In terms of severity, mild (grade I) SEL is often asymptomatic, while moderate (grade II) SEL is symptomatic in approximately 14.5% of cases. All severe (grade III) cases of SEL are symptomatic (1). A newly introduced MRI classification for lumbar spinal stenosis recognizes SEL as one of the three main contributors to central canal stenosis, alongside disc pathology and facet joint arthritis. This highlights the significance of SEL in the context of lumbar stenosis, which is often overshadowed by more common causes like disc herniation or facet joint degeneration. Early detection of SEL on MRI allows for better differentiation of stenosis causes, potentially leading to more tailored treatment strategies (9).

Despite these advancements, a study by Spinnato et al. revealed a concerningly low reporting rate of SEL (8%) among radiologists, regardless of their experience with MRI interpretation. This underreporting is thought to result from various factors, such as radiologists' expertise, incomplete clinical information during the diagnostic process, heavy workloads, other coinciding pathological findings that may explain symptoms, and differences in MRI protocols between institutions (10, 11).

The treatment approach for SEL is determined by the severity of symptoms. In cases where no acute neurological deficits are present, conservative management—such as discontinuing causative medications and implementing a structured weight loss plan—can be effective. For patients with obesity, weight loss or bariatric surgery has shown significant benefits. However, in cases with sudden onset of neurological deficits or bowel/bladder dysfunction, urgent surgical decompression may be required (3). Surgical intervention typically leads to favorable outcomes, with many patients experiencing full neurological recovery postoperatively (8).

## Conclusion

Early recognition of SEL in patients presenting with fluctuating neurological symptoms is crucial. Steroidal drugs must be avoided, and conservative measures should be taken once the diagnosis of spinal epidural lipomatosis is confirmed with a spinal MRI. Surgical treatment could be sought in case of failure of conservative trials or in severe abrupt neurological deficit.

## Data Availability

The original contributions presented in the study are included in the article/Supplementary Material, further inquiries can be directed to the corresponding authors.
